# Somatic hypermutation to counter a globally rare viral immunotype drove off-track antibodies in the CAP256-VRC26 HIV-1 V2-directed bNAb lineage

**DOI:** 10.1371/journal.ppat.1008005

**Published:** 2019-09-03

**Authors:** David Sacks, Jinal N. Bhiman, Kevin Wiehe, Jason Gorman, Peter D. Kwong, Lynn Morris, Penny L. Moore

**Affiliations:** 1 Centre for HIV and STIs, National Institute for Communicable Diseases (NICD) of the National Health Laboratory Service (NHLS), Johannesburg, South Africa; 2 Faculty of Health Sciences, University of the Witwatersrand, Johannesburg, South Africa; 3 Duke Human Vaccine Institute, Duke University School of Medicine, Durham, North Carolina, United States of America; 4 Vaccine Research Center, National Institute of Allergy and Infectious Diseases, National Institutes of Health, Bethesda, Maryland, United States of America; 5 Centre for the AIDS Programme of Research in South Africa (CAPRISA), University of KwaZulu Natal, Durban, South Africa; Miller School of Medicine, UNITED STATES

## Abstract

Previously we have described the V2-directed CAP256-VRC26 lineage that includes broadly neutralizing antibodies (bNAbs) that neutralize globally diverse strains of HIV. We also identified highly mutated “off-track” lineage members that share high sequence identity to broad members but lack breadth. Here, we defined the mutations that limit the breadth of these antibodies and the probability of their emergence. Mutants and chimeras between two pairs of closely related antibodies were generated: CAP256.04 and CAP256.25 (30% and 63% breadth, respectively) and CAP256.20 and CAP256.27 (2% and 59% breadth). Antibodies were tested against 14 heterologous HIV-1 viruses and select mutants to assess breadth and epitope specificity. A single R100rA mutation in the third heavy chain complementarity-determining region (CDRH3) introduced breadth into CAP256.04, but all three CAP256.25 heavy chain CDRs were required for potency. In contrast, in the CAP256.20/27 chimeras, replacing only the CDRH3 of CAP256.20 with that of CAP256.27 completely recapitulated breadth and potency, likely through the introduction of three charge-reducing mutations. In this individual, the mutations that limited the breadth of the off-track antibodies were predicted to occur with a higher probability than those in the naturally paired bNAbs, suggesting a low barrier to the evolution of the off-track phenotype. Mapping studies to determine the viral immunotypes (or epitope variants) that selected off-track antibodies indicated that unlike broader lineage members, CAP256.20 preferentially neutralized viruses containing 169Q. This suggests that this globally rare immunotype, which was common in donor CAP256, drove the off-track phenotype. These data show that affinity maturation to counter globally rare viral immunotypes can drive antibodies within a broad lineage along multiple pathways towards strain-specificity. Defining developmental pathways towards and away from breadth may facilitate the selection of immunogens that elicit bNAbs and minimize off-track antibodies.

## Author summary

Broadly neutralizing antibodies (bNAbs) develop in some HIV infected individuals, partly due to their complex evolutionary pathways that are characterized by extensive somatic hypermutation (SHM). Furthermore, bNAbs within a lineage may form a minor subset, amidst many strain-specific “siblings”, indicating that minor sequence differences between lineage members can significantly affect neutralization. Here, we define mutations that limit breadth in two “off-track” members of the CAP256-VRC26 bNAb lineage, and show that these occur with relatively high probability. A dominant autologous virus with a globally rare V2 sequence appears to have selected for an off-track antibody, providing a mechanism for the development of this antibody during infection. These data highlight the complex interdependencies between high levels of SHM and breadth, as mutations that neutralize autologous viruses may limit heterologous breadth. Consequently, strategies to increase SHM by repeated vaccinations will require careful antigen selection to focus the humoral response to globally common epitopes, limiting off-track responses.

## Introduction

Antiretroviral therapy has transformed HIV from a progressive fatal infection to a manageable chronic disease [1]. However, a preventative vaccine is urgently needed as drug resistance, access to medication and adverse side effects hamper the utility of antiretroviral drugs. Despite intensive research, strategies to induce protective immune responses by vaccination have achieved little success [2]. While neutralizing antibodies are elicited during HIV infection, the response is typically strain-specific. However, ~20% of individuals develop broadly neutralizing antibodies (bNAbs) which potently neutralize diverse global viruses *in vitro* and protect non-human primates from infection [3–8]. Therefore, understanding the mechanisms behind the elicitation and evolution of bNAbs is central to the development of an HIV vaccine.

During infection, the humoral response targets the HIV envelope (Env) glycoprotein which consists of three heavily glycosylated non-covalently linked gp41-gp120 protomers. While strain-specific antibodies recognize exposed and variable sites, bNAbs target relatively conserved and occluded sites, including the membrane proximal external region (MPER), gp120-gp41 interface, CD4-binding site (CD4bs), N332 supersite and a quaternary V1V2 epitope at the trimer apex [9]. Many V1V2 directed bNAbs have atypically long (>24 amino acids) third complementarity determining regions of the heavy chain (CDRH3) which provides access to glycan shielded and buried epitopes [10–14]. Additionally, bNAbs frequently exhibit unusually extensive somatic hypermutation (SHM), which suggests multiple rounds of affinity maturation. Understanding how these unusual features arise is critical to recapitulating this process by vaccination.

The degree to which SHM and affinity maturation occurs is influenced by HIV diversity and antigen load [15]. A number of longitudinal studies describing the “arms race” between viral quasispecies and bNAb lineages have provided insights into the evolution of breadth [16]. These studies have identified bNAb-initiating Envs that engage antibody precursors as well as emerging viral variants, which select bNAb intermediates [12,17–23]. We have extensively studied virus/antibody co-evolution in the superinfected CAP256 donor from whom 33 members of the CAP256-VRC26 V2-targetting bNAb lineage were isolated [11,12,22]. The evolution of this lineage was elucidated through analysis of longitudinal viral and antibody deep sequences from 59–206 weeks post infection [11,12,22]. Viruses from six weeks post infection are sensitive to neutralization by CAP256 lineage members but by 59 weeks post infection most viruses are resistant. Similar to other V2-directed bNAbs, the CAP256-VRC26 lineage members have very long (35–37 amino acid, aa) anionic and tyrosine-sulfated CDRH3s that recognize the electropositive V2 apex [24]. Modelling and mapping studies show that several residues within the CDRH3 are critical for epitope recognition, including the tyrosine sulfated YYD motif at the apex of the CDRH3 [24]. This motif is present in the unmutated common ancestor (UCA) of the CAP256-VRC26 lineage, 27 of the 33 lineage members as well as other V2-directed bNAbs such as PG9/PG16 [10,11]. In addition, the CDRH1 has been shown to stabilize the CDRH3, and residues in CDRH2 have been implicated in glycan recognition [25,26]. Within strands B and C of the viral Env protein, the CAP256-VRC26 lineage’s footprint includes residues N160, R166, D167, K168 and K169 and is similar to other V2 glycan bNAbs (PG9/PG16). The breadth, potency and extent of SHM of CAP256-VRC26 lineage members ranges from 2–63%, 0.003–5 μg/mL and 4.2–18%, respectively [11].

SHM, which is mediated by activation‐induced cytidine deaminase (AID), is essential for breadth as bNAb germline revertants generally lack substantial binding and neutralization [10,27–30]. Mutations that are selected during affinity maturation may increase antibody-antigen affinity directly at the paratope or indirectly by improving stability [31,32]. SHM is a semi-random process as AID hot-spots (WRCH/Y, W = A/T, R = A/G and H = A/C/T) and cold-spots (SYC, S = C/G and Y = C/T) are distributed throughout the antibody variable regions. Consequently, mutations associated with breadth are unlikely to occur if they are in cold-spots and/or require more than one nucleotide change [33,34]. Additionally, neutral mutations can accumulate via co-selection and constitutive background mutational noise [32]. Although increased SHM is generally associated with neutralization breadth, exceptions exist within the CAP256-VRC26 and other bNAb lineages. We have previously described these non-broad but highly mutated CAP256 antibodies as “off-track” [22]. Such off-track antibodies have been recovered from bNAb lineages in several individuals. 1AZCETI5 is a clonal member of the CD4bs CH103 bNAb lineage, and shares VDJ genes, CDRH3 length and extent of SHM with bNAb CH103, but the former only neutralizes autologous viruses [18]. The PGDM1400 bNAb, a member of the V2 glycan targeting PGDM lineage with 83% breadth has similar genetic properties to PGDM1406, but the latter exhibits limited breadth (6%) [35]. Similarly, within the PGT121 N332 targeting bNAb lineage are two heavy chains (HC) (22H and 6H) that are related to broad members but exhibit no neutralization [36]. As monoclonal antibody isolation methodologies are designed to recover bNAbs, the proportion of off-track antibodies in bNAb lineages is unknown. However, the finding that 40% of related antibodies recovered by flow cytometry from the donor of the CD4bs bNAb, VRC-PG04, were off-track, indicates that such antibodies may be common [37].

Collectively, studies into co-evolution and bNAb targets have identified candidate antigens to initiate antibody lineages and guide the development of breadth. Indeed, native-like Env trimers administered in combination or sequentially elicits tier 2 neutralizing antibodies in mice and rabbits, providing a strong rationale to test similar immunogens in people [38–42]. Importantly, the mutations crucial for breadth and potency as well as those that confer an off-track phenotype in bNAb lineages are largely unknown. Identification of these mutations will inform the design of vaccine antigens that elicit breadth but dampen the evolution of off-track responses. The high degree of sequence identity between CAP256-VRC26 bNAbs and their off-track relatives, the richly populated lineage and the availability of contemporaneous viral sequences is an ideal opportunity to compare off-track antibodies with bNAbs to determine why these antibodies lack breadth.

Here we define key residues that restrict neutralization breadth and potency in two off-track antibodies within the CAP256-VRC26 lineage. We found that in one off-track/bNAb pair, residues in each of the three CDRHs were necessary for potency and that a key breadth-conferring mutation in the CDRH3 was highly improbable. In the second pair, we show that the CDRH3 was completely responsible for modulating neutralization and that the mutations leading to the off-track phenotype were relatively probable. Lastly, through epitope mapping we identified viruses that guided the evolution of an off-track antibody toward a globally rare immunotype. This study highlights the stochastic nature of SHM and shows that breadth-restricting mutations typically arise with a greater probability than breadth-conferring mutations. This work provides insights into the evolution of breadth and mature strain-specificity, informing the design of immunogens that minimize the elicitation of off-track mutations while guiding evolution to globally conserved sites.

## Results

### Neutralization breadth is mediated by the CAP256-VRC26 antibody heavy chain

**Fig 1:**
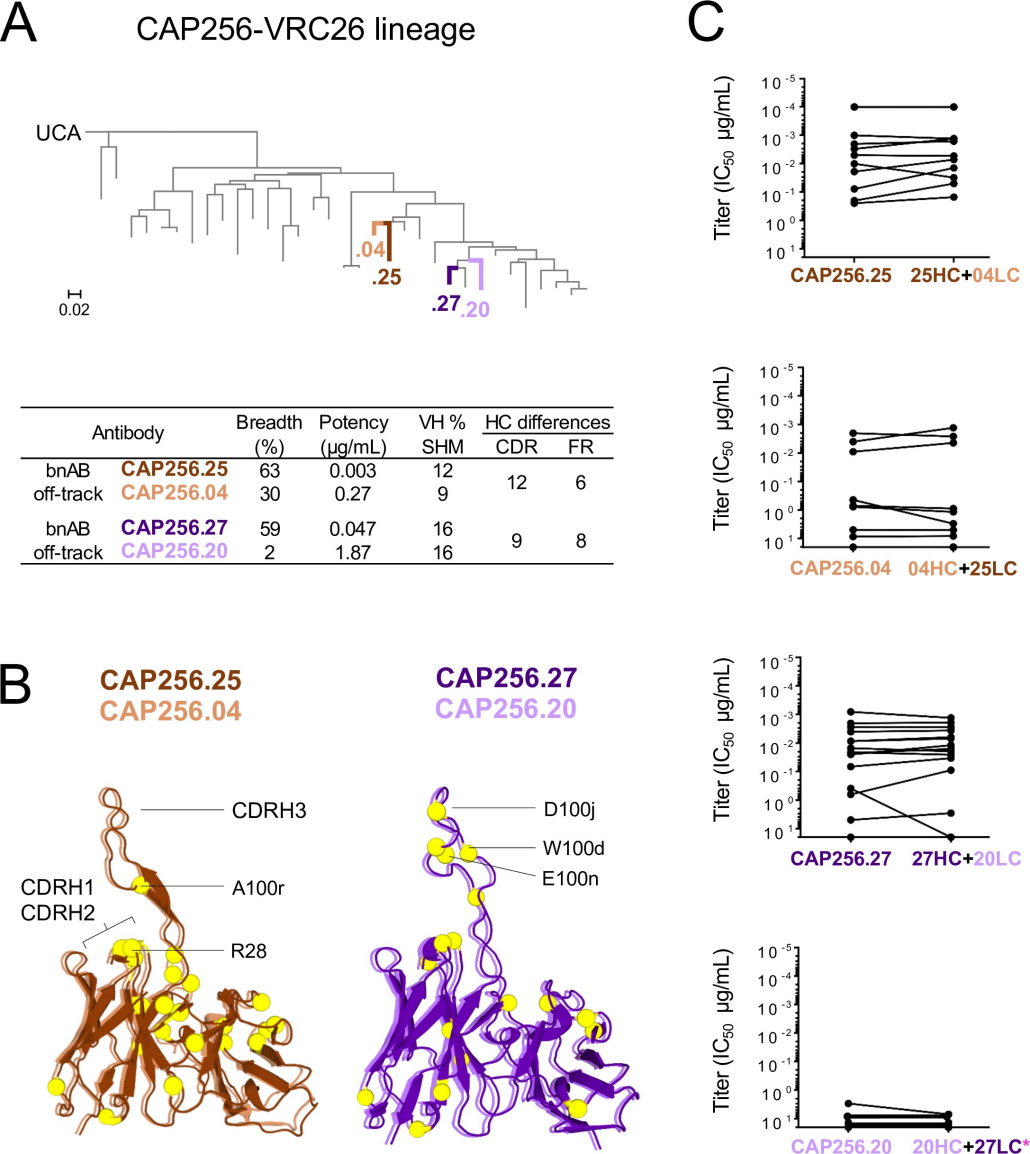
The HC mediates the off-track phenotype. (A) Maximum likelihood phylogenetic tree, rooted on the CAP256.UCA, with 33 clonally related VH sequences of monoclonal antibodies isolated from donor CAP256. Highlighted are CAP256.04 (orange), CAP256.25 (brown), CAP256.20 (light purple) and CAP256.27 (purple). Neutralization breadth, potency, VH SHM (related to CAP256.UCA) and the number of amino acids that differ between the CDR and FR of each pair are tabulated below. Breadth and potency (mean IC_50_) was calculated from a panel of 46 heterologous viruses [11]. (B, left) The amino acid sequence of CAP256.04 (orange) was fitted to a structure of CAP256.25 and CAP256 34-week trimer in Swiss-PdbViewer (v4.1.0), aligned in PyMOL (v2.0.2) and displayed as cartoons. (B, right) The amino acid sequences of CAP256.27 (purple) and CAP256.20 (light purple) were fitted to CAP256.25 in Swiss-PdbViewer (v4.1.0) and aligned in PyMOL (v2.0.2). The amino acids that differ between each bNAb and matched off-track antibody are represented as yellow spheres. (C) The LCs were exchanged between each bNAb/off-track pair and neutralization (IC_50_, μg/mL) was tested against (n = 16*) pseudoviruses.

To determine the genetic determinants that controlled the off-track phenotype, we selected two bNAbs with high sequence identity to two off-track antibodies. Off-track antibodies were defined as having low neutralization breadth despite their capacity to neutralize early autologous viruses [12], extensive sequence maturation and identity to related bNAb lineage members. The CAP256.25 bNAb was paired with the off-track antibody CAP256.04 and bNAb CAP256.27 was paired with the off-track antibody, CAP256.20. The two pairs of antibodies cluster together on a maximum likelihood tree (Fig 1A), consistent with our previous observations that antibodies with considerably different breadth may differ by relatively few amino acids [22]. The HC and light chain (LC) of CAP256.04 (30% breadth, 0.27 μg/mL potency, 9% SHM) both share 92% sequence identity with the HC and LC of bNAb CAP256.25 (63% breadth, 0.003 μg/mL potency, 12% SHM) (Fig 1A). Within the second pair, the HC and LC of CAP256.20 (2% breadth, 1.87 μg/mL potency, 16% SHM) shares 93% and 96% sequence identity with the CAP256.27 HC and LC (59% breadth, 0.047 μg/mL potency, 16% SHM), respectively (Fig 1A). A total of eighteen amino acids differ between the HC and 11 amino acids between the LC of CAP256.04 (orange) and CAP256.25 (brown) which are displayed as yellow spheres in an alignment of the two antibodies (Fig 1B). Seventeen amino acids (yellow spheres) differentiate the HCs and four the LCs of CAP256.27 (purple) and CAP256.20 (light purple) (Fig 1B).

As neutralization by members of the CAP256 bNAb lineage has been largely attributed to the HC [12], we first confirmed that the LC was not responsible for the off-track phenotype of CAP256.04 and CAP256.20. We generated chimeras between the HC and LC of each off-track/bNAb pair (25HC+04LC, 04HC+25LC and 27HC+20LC, 20HC+27LC) and tested their neutralization against both CAP256 infecting viruses (the primary and superinfecting viruses) and 14 heterologous viruses. The titers did not differ significantly (Wilcoxon signed rank test) between the chimeric antibodies and the natural antibody from which the HC was donated (Fig 1C). These data indicate that the LC has no impact on the neutralization breadth within these pairs, consistent with structural analyses showing no light chain contacts with the viral epitope [26], and we therefore focused on the HC in subsequent studies.

### Introduction of potency into CAP256.04 requires a combination of the CAP256.25 CDRHs

**Fig 2:**
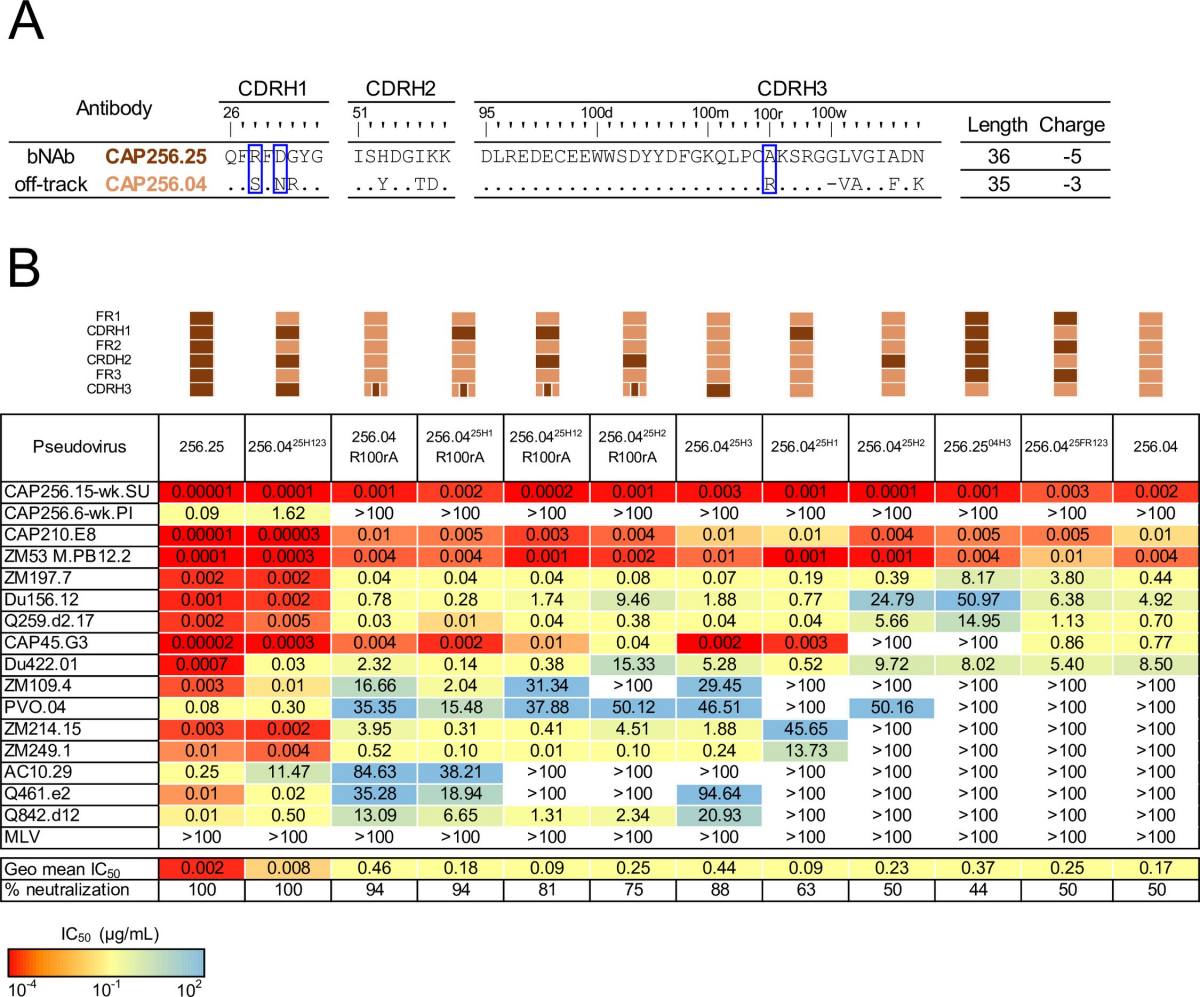
One mutation is required to introduce breadth into CAP256.04 while mutations in CDRH1, CDRH2 and CDRH3 are required for potency. (A) Comparison of the CDR sequences, including the length and charge of the CDRH3 of CAP256.04 (orange) and CAP256.25 (brown). The blue boxes highlights key off-track mutations. (B) Neutralization data using mutants and chimeras (indicated schematically above the table) between CAP256.25 and CAP256.04, with neutralization potency shown by color, as indicated in the key. Neutralization data represents the arithmetic mean titers (IC_50_, μg/mL) from at least two experiments.

To determine which regions of the HC restrict the breadth of CAP256.04, we constructed several chimeras and point mutants between CAP256.25 and CAP256.04, focusing initially on the CDRHs. The sequence variation between the CDRHs is shown in Fig 2A and includes charge and hydrophobicity differences between the two antibodies (R28S, D30N, G31R, H53Y, K57D, A100rR and N100ddF) and an insertion (G100w) in CAP256.25 that is absent from CAP256.04 (Fig 1B). We first transplanted all the CAP256.25 CDRHs (12 amino acid changes in total) into CAP256.04, creating CAP256.04^25H123^ (Fig 2B). Antibodies were tested against a panel of 16 viruses that were sensitive to CAP256.25 (geometric mean potency of 0.002 μg/mL against this panel), of which 8 were neutralized by CAP256.04 (with a 230-fold lower geometric mean potency of 0.17 μg/mL) (Fig 2B). The neutralization breadth and potency of the CAP256.04^25H123^ chimera (which neutralized all 16 viruses with a geometric mean potency of 0.008 μg/mL) matched that of CAP256.25 (0.002 μg/mL) (Fig 2B). In the reverse experiment, introducing the framework (FR) regions from CAP256.25 into CAP256.04 (CAP256.04^25FR123^) had no effect on breadth (Fig 2B). These data suggest that the mutations in the CDRs, rather than the FW regions restrict the breadth of CAP256.04.

To dissect the role of individual CDRHs, each was individually swapped between the two parental antibodies. CAP256.04^25H1^ neutralized two additional viruses (ZM214 and ZM249) that were resistant to CAP256.04 (Fig 2B). In addition, we observed virus-specific increases in potency for some sensitive viruses (with a 6-18-fold improvement for Du156, Q259, and Du422 and a 250-fold increase in potency for CAP45). In contrast, CAP256.04^25H2^ neutralized the same number of viruses as CAP256.04 (Fig 2B), though again slight virus-specific differences in potency were observed, with CAP45 becoming resistant to CAP256.04^25H2^ while PVO gained sensitivity (Fig 2B). The largest effect of a single CDRH swap was observed for the CDRH3 which differed by six amino acids. CAP256.04^25H3^ neutralized six additional viruses (ZM109, PVO, ZM214, ZM249, Q461 and Q842) (Fig 2B) although with similar potency to CAP256.04 (0.39 μg/mL and 0.17 μg/mL, respectively). This was confirmed in the reverse experiment, where the CAP256.04 CDRH3 was introduced into CAP256.25 (CAP256.25^04H3^) resulting in reduced breadth (Fig 2B). Overall, these data suggest that the breadth of the CAP256.04 off-track phenotype is modulated by the CDRH3, but potency is attenuated by a combination of all three CDRHs.

### Introduction of breadth into CAP256.04 requires a single improbable CDRH3 mutation

As the CAP256.25 CDRH3 conferred additional breadth into CAP256.04, we assessed which residue(s) were responsible for this effect (Fig 2A). Mutating CDRH3 residues that altered the charge (K102N) or aromaticity (F100ddA) of the CDRH3 failed to improve the neutralization of CAP256.04 (S1 Fig). In contrast, the R100rA mutation, which removed a positive charge from the CDRH3, improved the breadth of CAP256.04 from 50% to 94% of this panel, with seven viruses resistant to CAP256.04 becoming sensitive to CAP256.04 R100rA (Fig 2B). Interestingly, this single mutation recapitulated the breadth introduced into CAP256.04 by the entire CAP256.25 CDHR3. The reverse mutant, CAP256.25 A100rR similarly resulted in a substantial loss of breadth (S1 Fig). The combination of R100rA with the CAP256.25 CDRH1 did not confer additional breadth beyond that introduced by R100rA (Fig 2B) and, surprisingly, the addition of the CDRH2 to either CAP256.04 R100rA or CAP256.04^25H1^ R100rA reduced the breadth of these antibodies (Fig 2B).

**Fig 3:**
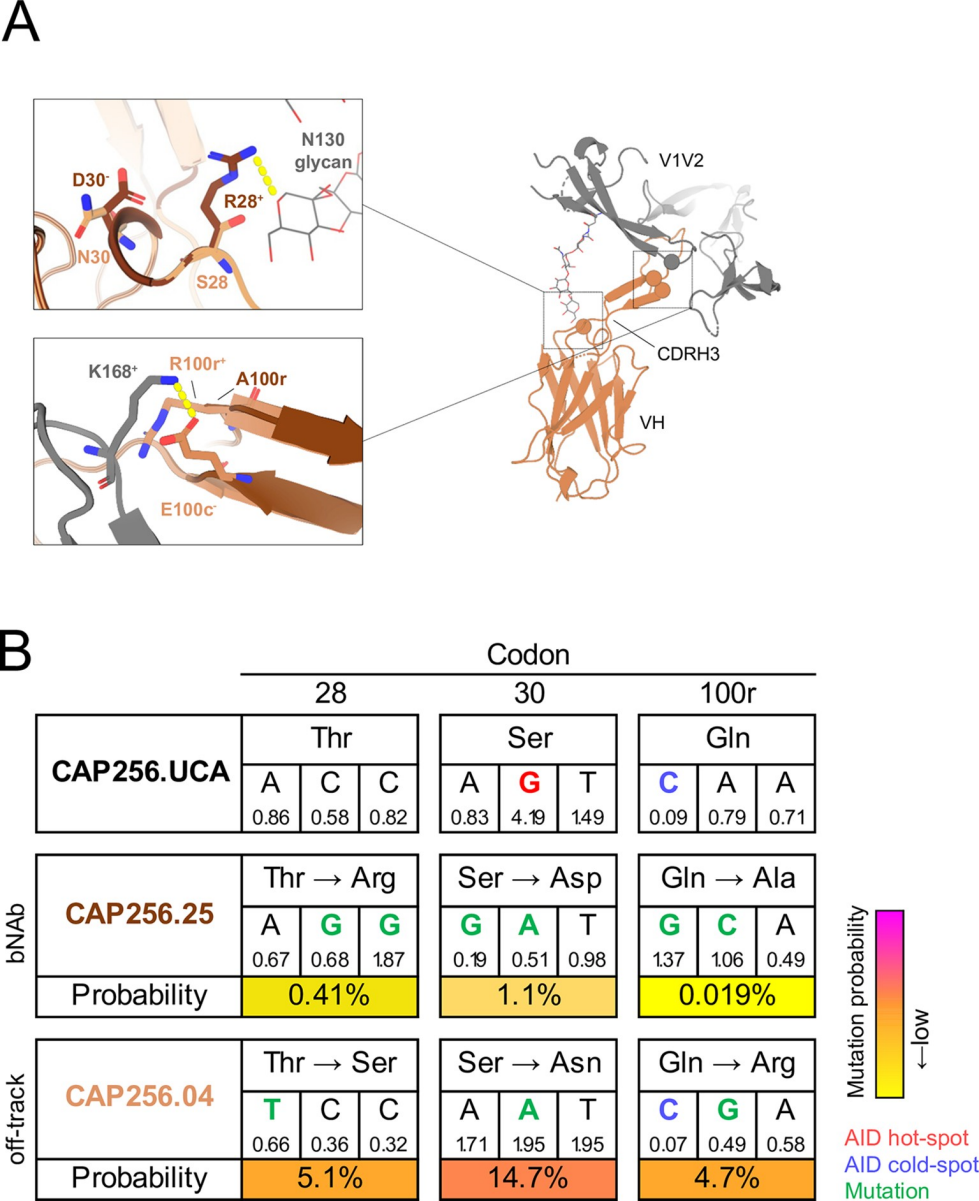
The structural effect and probabilities of the CAP256.04 off-track mutations S28, N30 and R100r. (A) The amino acid sequence of CAP256.04 (orange) was fitted to a model of CAP256.25 (brown) and the V1V2 of trimeric CAP256 34-week Env (grey, [26]) in Swiss-PdbViewer (v4.1.0) and visualized in PyMOL (v2.0.2). The top inset shows the CAP256.25 CDRH1 D30 (brown) residue and a hydrogen bond between CAP256.25 CDRH1 residue R28 (brown), and the glycan at N130. The bottom inset displays CAP256.04 R100r (orange) in proximity to V2 K168. Symbols ^+^ and ^-^ denote positive and negative charge, respectively. (B) The germline (T28, S30 and Q100r) and mature amino acid sequences (CAP256.25 T28R, S30D and Q100rA, and CAP256.04 T28S, S30N and Q100rR) at codons 28, 30 and 100r are displayed with the nucleotide sequence of each codon. Blue and red text indicates AID cold-and hotspots, respectively. Green text refers to mutations and yellow to pink shading indicates low and high probability mutations, respectively. The mutability scores are shown below each nucleotide.

To model the interactions between V1V2 and CAP256.04 or CAP256.25, we fitted the sequence of CAP256.04 to the crystal structure of CAP256.25 in complex with the CAP256-34 week trimer which shares key contact residues for the CAP256-VRC26 lineage, including N160, R166, D167, K168 and K169 with CAP256.15-wk SU (Fig 3A), and which likely triggered the lineage [22]. The model showed that the N130 glycan projects from the trimer and is situated proximal to the CDRH1. The CAP256.25 R28 residue was predicted to hydrogen bond with the N130 glycan while CAP256.04 S28 was not predicted to interact with this glycan. Surprisingly, the introduction of S28R to CAP256.04 R100rA and CAP256.04^25H3^ (S1 Fig) decreased the breadth of these antibodies by five and four viruses, respectively, although the potency remained unchanged. Together with R100rA, the combination of S28R and N30D increased the breadth compared to S28R alone, but two viruses (PVO.04 and AC10.29) remained resistant to neutralization (S1 Fig). The conformation of the CDRH3 is partly supported by the intermolecular interactions between this loop and the CDRH1 and CDRH2 [25,26]. Consequently, the interaction between R28 and the CDRH3 may shift the CDRH1 toward the N130 glycan, which may be countered by the N30 residue (Fig 3A, top panel). Furthermore, the model showed that 100r was positioned in close proximity to the viral residue K168, and that K168 was hydrogen bonded to the CDRH3 residue E100c (Fig 3A, bottom panel). Therefore, due to electrostatic repulsion, R100r likely displaces K168, disrupting the interaction between K168 and E100c. In contrast, CAP256.25 has a small, uncharged Ala at position 100r which would not obstruct the adjacent bond. This suggests that an R100rA mutation may reduce electrostatic repulsion and therefore increase antigen affinity, providing a mechanism for the enhanced breadth associated with this mutation.

To estimate the probability of the occurrence of mutations prior to selection that are associated with the off-track phenotype, we utilized the ARMADiLLO software [34]. We first assessed the probability of the CDRH1 T28 residue (found in the CAP256.UCA) mutating to either 28R (as in CAP256.25) or 28S (in CAP256.04, Fig 2A). We found that the CAP256.25 T28R mutation was improbable (0.41%), requiring two base changes (ACC to AGG), whereas T28S (in CAP256.04) was ten times more probable (5.1%), requiring only one change (ACC to TCC, Fig 3B). Similarly, two base changes were necessary for the improbable (1.1%) mutation S30D (in CAP256.25), whereas S30N (in CAP256.04) was a probable (14.7%) event as it occurred in an AID hot-spot. The combination of T28R and S30D (in CAP256.25), which was necessary for neutralization when transplanted into CAP256.04, was a highly improbable event (0.004%, Fig 3B). Furthermore, the CDRH3 Q100rA mutation, that confers breadth to CAP256.25, was predicted to occur very rarely with a 0.019% probability, requiring two base changes (CAA to GCA), one of which was in an AID cold-spot (Fig 3B). In contrast, we found that Q100rR that results in the off-track phenotype was predicted to occur with a much higher probability of 4.7%, requiring only a single nucleotide change that was not located within an AID cold-spot (Fig 3B). Furthermore, the improbability of the evolution of T28R, N30D and Q100rA was reflected in next-generation sequences of the CAP256 lineage [22,43], where T28R, S30D and Q100rA together accounted for <2% of the sequences. Altogether, this suggests that a single relatively probable mutation in the CDRH3 primarily limits the neutralization breadth of CAP256.04.

### The CDRH3 alone modulates neutralization breadth and potency of the off-track antibody CAP256.20

**Fig 4:**
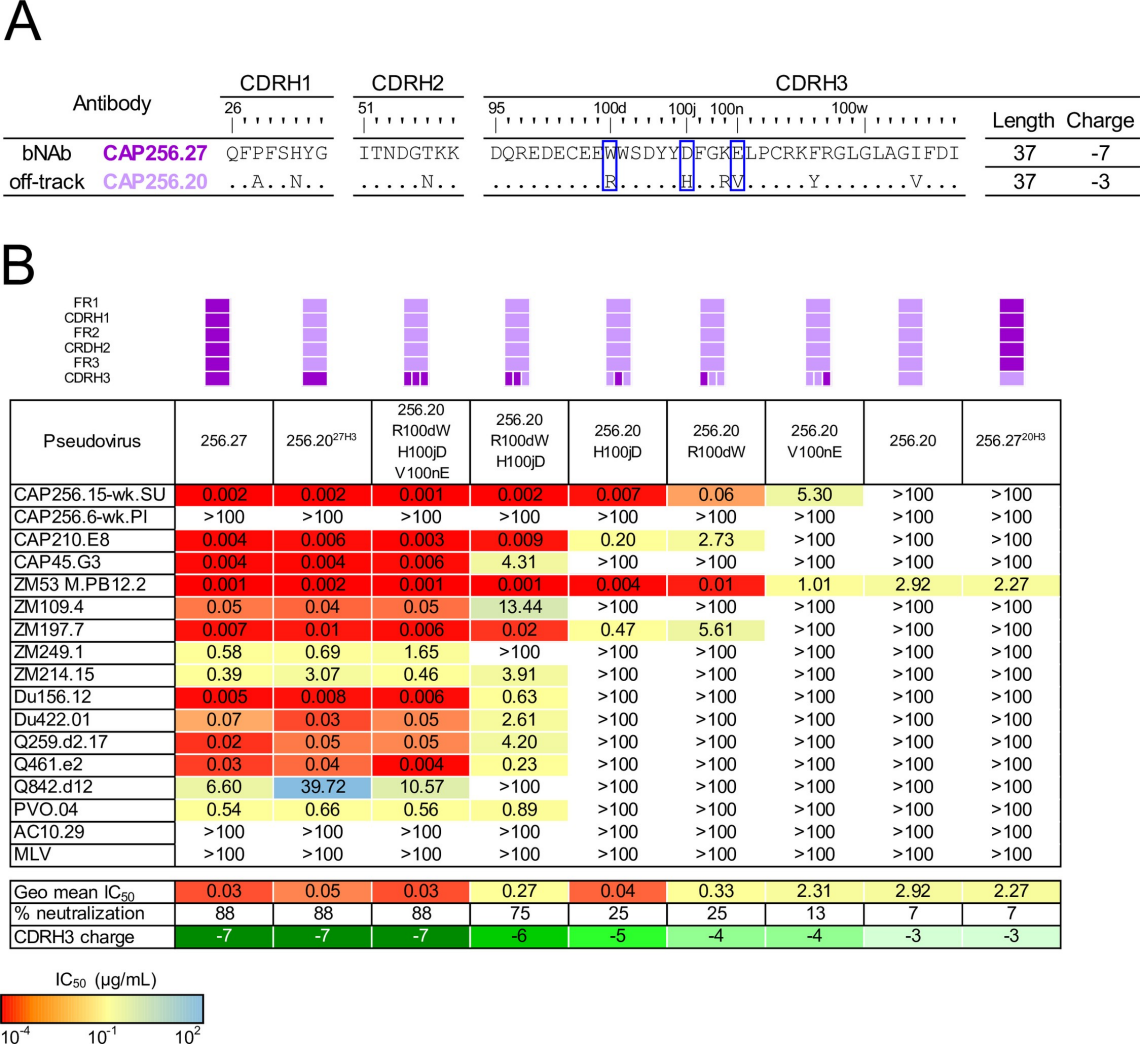
The CDRH3 alone restricts the neutralization breadth and potency of CAP256.20. (A) Comparison of the CDR sequences of CAP256.20 (light purple) and CAP256.27 (purple). The blue box highlights key off-track mutations. (B) Neutralization data using mutants and chimeras (indicated schematically above the table) between CAP256.27 and CAP256.20, with neutralization potency shown by color, as indicated in the key. Neutralization data represents the arithmetic mean titers (IC_50_, μg/mL) from at least two experiments.

Next, we sought to determine which sites were responsible for the off-track phenotype of CAP256.20 compared to the related bNAb, CAP256.27 (Fig 4A). Of the 6 amino acids that distinguish these CDRH3s, several were charge changes, with an overall CDRH3 charge of -7 and -3 in CAP256.27 and CAP256.20, respectively. First, we transplanted the CDRH3 from CAP256.27 into CAP256.20 (CAP256.20^27H3^) and tested this chimera for neutralization breadth. In contrast to the CAP256.04/.25 pair, transfer of only the CAP256.27 CDRH3 into CAP256.20 introduced both bNAb-like breadth and potency into the off-track antibody, increasing breadth from 1/16 to 14/16 viruses and potency from 2.92 to 0.05 μg/mL (Fig 4B). In the reverse experiment, replacing the CDRH3 of CAP256.27 with that of CAP256.20 (CAP256.27^20H3^) almost completely abrogated neutralization (Fig 4B).

To determine which of the six residues within the CDRH3 of 256.20 limited breadth, we focused on three residues which altered the electronegativity of the loop, R100d, H100j and V100n (Fig 4A), and mutated these sites alone and in combination. The V100nE mutation, which slightly decreased the charge of the CDRH3 only marginally improved neutralization breadth by one additional virus (the autologous superinfecting virus, CAP256.15-wk SU) (Fig 4B). R100dW also decreased the charge and increased breadth by an additional three viruses (CAP256.15-wk SU, CAP210 and ZM197). Similarly, introduction of an H100jD mutation conferred neutralization of the same three additional viruses but with greater potency than R100dW (geometric mean potency of 0.04 compared to 0.33 μg/mL, respectively). The combination of R100dW and H100jD increased potency and breadth to a total of 12/16 viruses, while V100nE, R100dW and H100jD together introduced the same bNAb-like broad neutralization and potency as the entire CDRH3 transplant (Fig 4B). In the reverse experiment, mutating positions 100d, 100j and 100n in the CDRH3 of CAP256.27 to match the sequence of CAP256.20 knocked out neutralization against previously sensitive viruses (S2 Fig). Therefore, of the 17 residues that differentiate the HC of CAP256.27 and CAP256.20 only three amino acids, all within the CDRH3, are responsible for limiting the breadth of CAP256.20.

**Fig 5:**
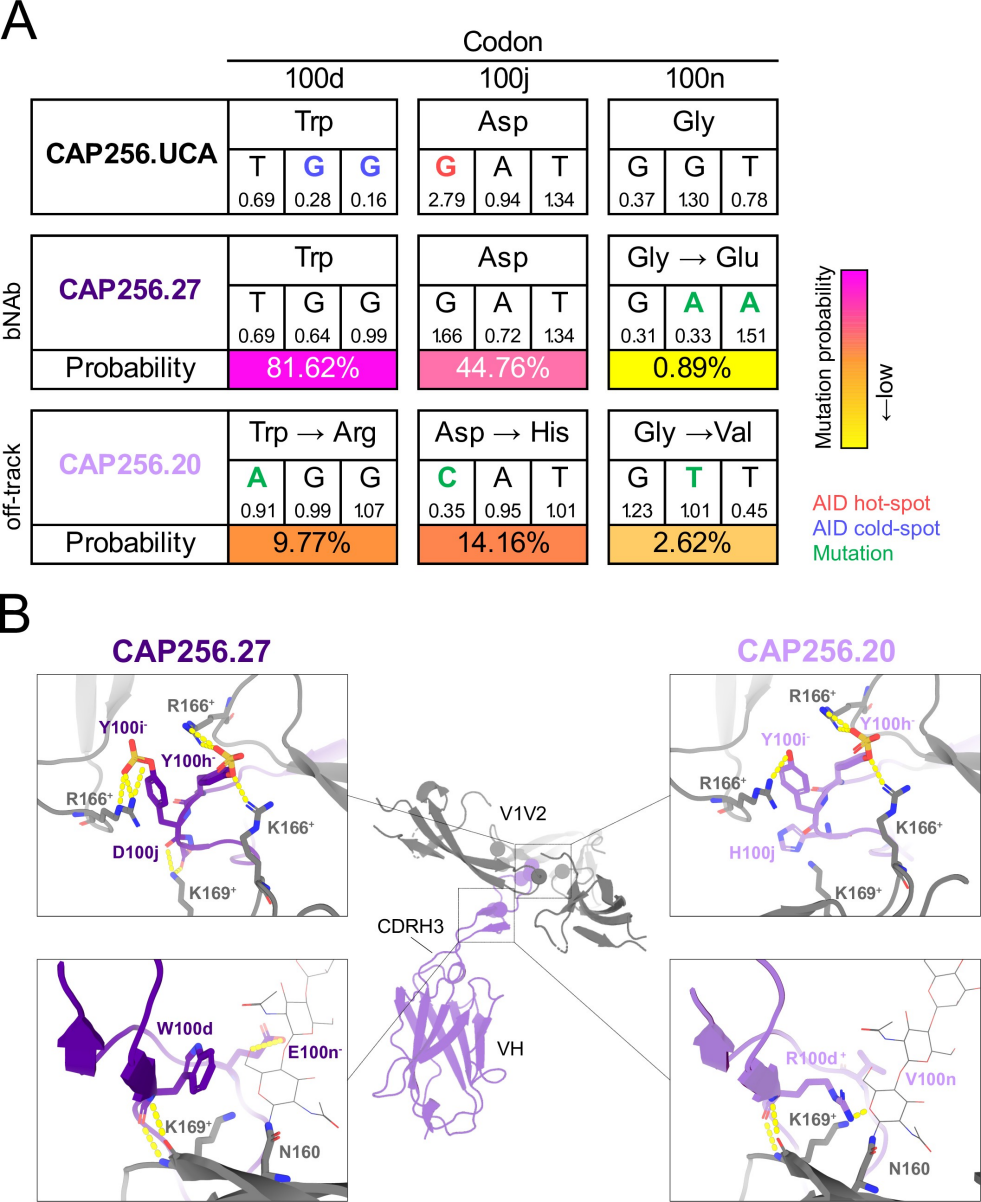
The probability and structural effect of the CAP256.20 off-track mutations W100dR, D100jH and G100nV. (A) The germline (W100d, D100j and G100n) and mature nucleotide and amino acid sequences (CAP256.27 W100d, D100j and G100nE and CAP256.20 W100dR, D100jH and G100nV) at codons 100d, 100j and 100n are displayed. Blue and red text indicate AID cold- and hot-spots, respectively. Green text refers to mutations, and yellow to pink shading indicates low and high probability mutations, respectively. The mutability scores are below each nucleotide. (B) The sequence of CAP256.20 (light purple) and CAP256.27 (purple) were fitted to a structure of CAP256.25 and trimeric CAP256 34-week Env [26] in Swiss-PdbViewer (v4.1.0) and aligned and visualized in PyMOL (v2.0.2). The top panels shows the interaction between the CDRH3 YYD motif (purple/light purple) and the V2 epitope (grey). The bottom panel displays the CAP256.27 residues W100d and E100n (purple) and CAP256.20 R100d and V100n (light purple) in proximity to K169, ^+^ and—denotes positive and negative charge, respectively.

Comparison of the probabilities of the breadth-conferring and restricting mutations between CAP256.27 and the CAP256.UCA showed that the G100nE bNAb mutation was improbable (0.89%), since two base changes were required (Fig 5A). In contrast, the W100dR, D100jH and G100nV mutations that took CAP256.20 off-track are highly probable (10%, 14% and 3%, respectively), as only single base changes were necessary, with the H100j mutation in an AID hot-spot (Fig 5A). The probability of retaining all three mutations that confer breadth (W100d, D100j and G100n) in the absence of selection is 0.33%. Overall, the relatively higher probability of these mutations which restrict breadth highlights the potential ease with which members of bNAb lineages may mature along undesirable pathways.

To explore the basis of these results, we fitted the amino acid sequences of CAP256.20 and CAP256.27 HCs to the crystal structure of CAP256.25 HC in Swiss-PdbViewer (v4.1.0), and then aligned this to a CAP256-34 week trimer structure (Fig 5B, [26]). The YYD motif of CAP256.27 was predicted to hydrogen bond with two K166 residues on two protomers and R166 and K169 on the remaining protomer (Fig 5B, top left). The mutations in CAP256.20 likely disrupt these interactions by placing a positive charge (H100j) proximal to K169 and possibly preventing sulfation of the preceding Tyr [44], which would knock out contacts with K166 (Fig 5B, top right).

Furthermore, the hydrogen bond between E100n, present in CAP256.27, and the N160 glycan was disrupted by the CAP256.20 V100n substitution (Fig 5B, bottom left). In addition, the CAP256.20 R100d mutation, places a positive charge next to K169 resulting in electrostatic repulsion (Fig 5B, bottom right) and possibly preventing the backbone interactions between these residues. Therefore, key interactions between the highly electronegative CDRH3 of CAP256.27 (-7) and the electropositive V2 epitope are abrogated by the more electropositive CDRH3 of CAP256.20 (-3), resulting in the off-track phenotype.

### CAP256.20 was dependent on a globally rare Q169 immunotype

**Fig 6:**
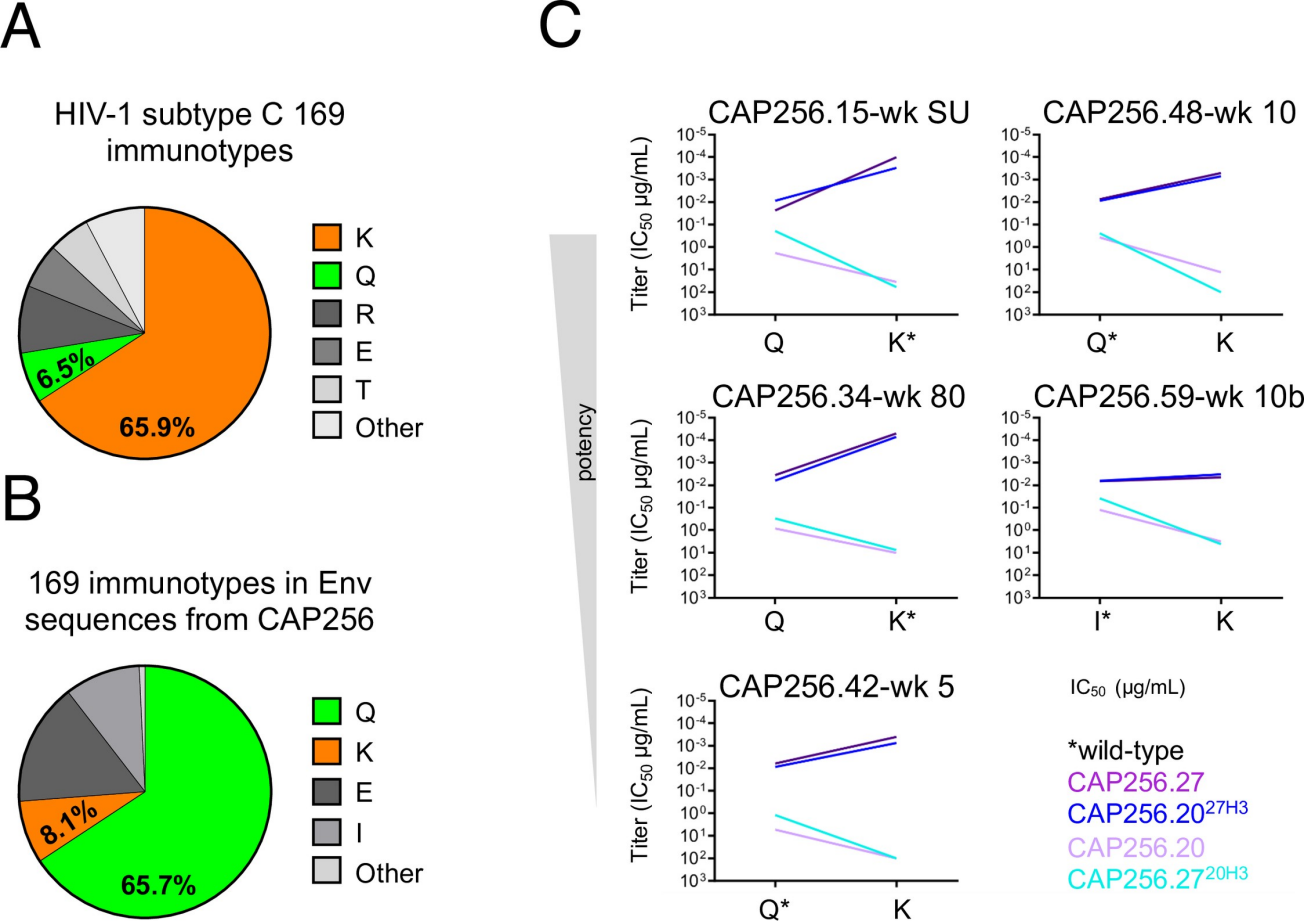
The off-track antibody CAP256.20 neutralizes a globally rare 169Q immunotype that was common in donor CAP256. (A) The K169 (orange) immunotype is common in globally circulating viruses, (B) while 169Q (green) predominates in CAP256 across most time points. (C) Slope graphs displaying the change in neutralization titer (IC_50_) of CAP256.20 (light purple), CAP256.27 (purple), CAP256.20^27H3^ (blue) and CAP256.27^20H3^ (cyan) against K169 and 169Q autologous viruses.

We were interested in determining which viral immunotypes (or epitope amino acid variants) drove CAP256.20 away from breadth. The K169 immunotype, which forms part of the CAP256 epitope, is fairly conserved within subtype C viruses (65.9%) (www.hiv.lanl.gov) (Fig 6A) [12]. In contrast, the 169Q immunotype is present in only 6.5% of subtype C viruses (Fig 6A) but dominated the viral population in donor CAP256 across 21 of 28 time points from six to 206 weeks post infection (Fig 6B and S3A Fig). We hypothesized that this globally rare immunotype, which predominated in CAP256, contributed to the evolution of CAP256.20. Introduction of 169Q mutations into eleven heterologous viruses slightly improved the neutralization of four viruses by CAP256.20 (S3B Fig), suggesting a preference of CAP256.20 for 169Q, though other viral determinants clearly contribute to neutralization sensitivity/resistance.

As autologous viruses, rather than heterologous viruses, select mutations during SHM, we determined the sensitivity of autologous viruses containing either a 169Q or 169K immunotype to neutralization by CAP256.20 and CAP256.27. We identified two viruses that naturally contained a 169Q (CAP256.42-wk 5 and CAP256.48-wk 10), and were sensitive to CAP256.20 and CAP256.27^20H3^. Introduction of Q169K mutations into these viruses knocked out neutralization by these antibodies, and their matched CDRH3 chimeras (Fig 6C). In the reverse experiment, introduction of a 169Q into three autologous viruses (CAP256.34-wk 80, CAP256.59-wk 10b and CAP256.15-wk SU, containing 169K/I and resistant to CAP256.20 and CAP256.27^20H3^) resulted in increased sensitivity to these antibodies (Fig 6C). In all five viruses, the 169K immunotype was associated with increased neutralization potency by CAP256.27, CAP256.20^27H3^ and the additional broad CAP256.20 mutants and chimeras (S3C Fig), consistent with previous studies [11,12]. These data indicate a CDRH3-mediated preference of CAP256.20 for the 169Q immunotype, which limited the evolution of breadth in this antibody.

## Discussion

A major focus of HIV vaccine design is based on a deep understanding of the development of bNAbs during HIV infection. Studies of HIV/antibody co-evolution have provided a template for the maturation of breadth that is now the basis of B-cell lineage vaccine strategies [12,17–23]. However, much less is known about the maturation of antibodies within bNAb-containing lineages that fail to acquire breadth. We have previously shown that these include both “dead-end” antibodies (that fail to acquire breadth and exhibit low SHM), and “off-track” antibodies that acquire substantial SHM, but little breadth, and are the focus of this study. Here we used previously identified strain-specific “off-track” antibodies that are closely related to broad members of the CAP256-VRC26 bNAb lineage to probe the genetic and viral contributors to their maturation [22]. In two pairs, we identified key breadth-restricting mutations, and defined the probabilities of their occurrence. Furthermore, we show the preferential neutralization of a globally rare immunotype by CAP256.20 impeded the evolution of breadth, providing a mechanism for the development of off-track responses in infection. Together these data provide insights into the challenges associated with driving maturation of antibodies towards breadth, a central question in HIV vaccine design.

Studies of bNAb lineages have highlighted the substantial plasticity in their maturation, enabling the development of breadth through multiple pathways [11,12,21,43]. This suggests that immunization strategies promoting high levels of SHM along diverse pathways may enhance bNAb development. Our data suggests that similarly, the development of off-track antibodies can occur by multiple pathways, and through the acquisition of very few mutations. Indeed, within the CAP256.20/27 pair, only three mutations were sufficient to divert CAP256.20 away from both breadth and potency. Furthermore, the enhanced breadth of CAP256.04 R100rA, which contains a single CDRH3 mutation, in contrast to the six mutations in CAP256.04^25H3^, suggests substantial mutational “noise”. Additionally, we found an increase in breadth when the CAP256.25 CDRH2 or single CDRH1 mutations were introduced into CAP256.04. These data confirm that extensive SHM does not necessarily result in breadth and suggests that many mutations do not impact or negatively affect breadth.

The maturation of bNAbs towards breadth includes a requirement for functionally relevant but improbable mutations [34]. Consistent with previous studies, we find that the key breadth-conferring mutations in the CAP256-VRC26 lineage, such as Q100rA and G100nE, are highly improbable [34] and that mutations that restricted breadth were in many cases relatively probable. Interestingly, the two pairs of CAP256 antibodies provide distinct examples of how the probability of mutations could shape on- versus off-track maturation. In CAP256.20/27, the potential for high probability breadth-limiting mutations (e.g. W100dR, D100jH and G100nV) is evident in pulling the B-cell off-track. In contrast, in the CAP256.04/25 pair, an important improbable mutation associated with breadth, Q100rA, represents a potential bottleneck that CAP256.25 has overcome to achieve breadth, but CAP256.04 has not. The more probable R100r mutation is also present in broader lineage members, suggesting compensatory mechanisms and that multiple pathways to breadth exist. Together these data indicate that, in some instances, the pathway to the off-track phenotype offers less “resistance” compared to the requirement for highly improbable mutations associated with breadth, and will require careful selection of immunogens to avoid this phenotype [34].

Several studies, including our previous work in the CAP256-VRC26 lineage, have shown how exposure to diverse viral variants contributes to the development of breadth [11,12,22]. This study extends this work to define the mechanisms that select the off-track phenotype within a single lineage. The enrichment of the globally rare 169Q immunotype in CAP256 and the preferential neutralization of this immunotype by CAP256.20, implicates 169Q autologous viral variants in the evolution of this off-track antibody. Early CAP256-VRC26 lineage members (CAP256.01 and CAP256.24) and other off-track antibodies (CAP256.12 and CAP256.13) are unable to neutralize 169Q viruses. However, most lineage members, especially those with breadth, are able to neutralize this immunotype, though to a lesser extent than K169 viruses. This indicates that whereas most lineage members tolerate 169Q, CAP256.20 is uniquely reliant on the globally rare Q169 immunotype, explaining the strain-specificity of this antibody. Structurally, this may be a consequence of the reduced dependence of bNAbs on specific side chain residues within the epitope, compared to an increased dependence of off-track antibodies such as CAP256.20 on such side chains. These data highlight the fact that evolution towards breadth, a desirable outcome from a vaccinology perspective, is distinct from antibody maturation to counter circulating autologous viral variants.

This study provides evidence that affinity maturation to counter globally rare viral immunotypes can drive antibodies within a broad lineage away from breadth. As with the maturation of breadth, off-track antibodies can develop through multiple evolutionary pathways. Furthermore, limited breadth despite high levels of SHM can occur by the introduction of few, but relatively probable, mutations. The inherently stochastic nature of affinity maturation may make avoiding off-track antibodies challenging. Furthermore, additional research is needed to determine if the expansion of ‘off-track’ B-cell lineages prevents or limits the maturation of bNAbs. However, defining pathways towards and away from breadth will facilitate the selection of immunogens that elicit bNAbs and minimize off-track antibodies. Our data suggests that by selecting sequential immunogens that present globally conserved epitopes, the elicitation of off-track antibodies can be minimized. As immunization strategies improve to allow targeting of specific antibody residues, these data inform the design of immunogens to elicit V2-directed bNAbs.

## Materials and methods

### Ethics statement

CAP256 is a participant enrolled in the CAPRISA 002 Acute Infection study, established in 2004 in Kwa-Zulu Natal, South Africa. The CAPRISA 002 Acute Infection study was reviewed and approved by the research ethics committees of the University of KwaZulu-Natal (E013/04), the University of Cape Town (025/2004) and the University of the Witwatersrand (MM040202). CAP256, an adult, provided written informed consent. The specificity of her plasma has been described, monoclonal antibodies isolated and autologous Env evolution characterized [11,12,22,45,46].

### Overlapping PCR and site-directed mutagenesis

Exchanging the CDRH3s between monoclonal antibodies was achieved with a two-step overlapping PCR. The CDRH3s were amplified with the AccuPrime Pfx DNA Polymerase and reaction mix (Thermo) with primers that were complementary to the recipient antibody. The product was gel extracted (1%, 1x TAE) and purified with the QIAquick Gel Extraction Kit (Qiagen) and used as the mutagenesis primer for the second PCR with the QuikChange Lightning Multi Site-Directed Mutagenesis Kit (Stratagene). Genes with CDRH1, CDRH2 and CDRH3 exchanges were synthesized (GenScript) and excised with Age1 and Sal1 (Thermo) according to the manufacture’s recommendation. Heavy chain fragments were separated in an agarose gel (1% 1x TAE) and ligated (Roche) into the CMVR expression vector as per the manufacturer’s protocol. Mutations in both antibody and viral Env genes were introduced by site-directed mutagenesis with the QuikChange Lightening Multi Kit (Stratagene) per the manufacturer’s instructions and plasmid sequences were confirmed by Sanger sequencing with the ABI PRISM Big Dye Terminator Cycle Sequencing Ready Reaction kit (Applied Biosystems, Foster City, CA) and resolved on the 3500 genetic analyzer.

### Cell lines

The TZM-bl cell-line was obtained from the AIDS Research and Reference Reagent Program and the 293T cell-line was obtained from Dr. George Shaw (University of Pennsylvania, Philadelphia, PA). The cell-lines were cultured at 37°C (5% CO_2_) in Dulbecco’s Modified Eagle Medium (DMEM) supplemented with 10% heat-inactivated foetal bovine serum (FBS), 50 μg/mL gentamicin (Sigma) and 25 mM HEPES, at confluency monolayers were disrupted with 0.25% trypsin and 1 mM EDTA (Sigma). The 293F cell-line (Life Technologies) was maintained in serum and antibiotic free FreeStyle 293 Expression media at 37°C (10% CO_2_) in an orbital shaker (130 rpm).

### Pseudovirus production

Cloned Envs and pSG3ΔEnv backbone plasmids (NIH AIDS Research and Reference Reagent Program) were co-transfected into 293T cells with 1:3 PEI MAX transfection reagent (Polysciences). Following 48 hours, filtered pseudovirus supernatants were adjusted to 20% FBS and stored at -80°C.

### Antibody expression

Equal quantities of antibody heavy and light chain plasmids were co-transfected into 293F cells using PEI MAX. Monoclonal antibodies were purified from cell-free supernatants after six days using protein A affinity chromatography. Concentration and buffer exchange (1x PBS) was completed with Vivaspin concentrators (Sartorius).

### Neutralization assays

Neutralization was measured as previously described by a reduction in luciferase gene expression after single-round infection of TZM-bl cells with Env-pseudotyped viruses [47,48]. Titers were calculated as the reciprocal antibody dilution (IC_50_) causing 50% reduction of relative light units (RLU).

### Models

The crystal structures of CAP256.04 (PDB: 4ORG) was aligned to the crystal structure of CAP256.25 and CAP256-34 week trimer in PyMOL 2.0.2. No structures were available for CAP256.20 and CAP256.27, therefore the amino acid sequences of these antibodies were fitted to the crystal structure of CAP256.25 in Swiss-PdbViewer 4.1.0 and aligned in PyMOL. Protein model representations were created with Swiss-PdbViewer v4.1.0 and PyMOL v2.0.2. Graphs were created in GraphPad Prism 6, sequence alignments were made in BioEdit v7.2.5 and phylogenetic trees were constructed in MEGA v6.06.

### Probability of antibody mutations

The Antigen Receptor Mutation Analyzer for Detection of Low Likelihood Occurrences (ARMADiLLO) program estimates the probability of amino acid substitutions prior to antigenic selection [34]. Briefly, 10^4^ simulated mature sequences per site of interest are generated with the S5F mutability and substitution models through comparison of the nucleotides sequences of the CAP256.UCA and a given mature lineage member [33]. The probability of particular amino acid substitutions is then estimated by the frequency of the observed site in the set of simulated sequences. Here, a mutation of <2% probability is classified as “improbable” as this reflects a frequency ≤2 B-cells per germinal centre harbouring that particular mutation [34]. Probabilities of combinations of mutations are derived from the products of individual probabilities.

## Supporting information

S1 Fig Neutral and deleterious mutations in the CAP256.25/04 pair. Neutralization data using mutants (indicated schematically above the table) between CAP256.25 and CAP256.04, potency indicated as per the key. Arithmetic mean titers (IC_50_, μg/mL) from at least two experiments are reported.
(TIF)

S2 Fig Off-track conferring mutations knock out CAP256.27 neutralization. Neutralization data using mutants (indicated schematically above the table) between CAP256.27 and CAP256.20, potency indicated as per the key. Arithmetic mean titers (IC_50_, μg/mL) from at least two experiments are reported.
(TIF)

S3 Fig 169Q immunotype predominates in CAP256 and CAP256.20 exhibits weak neutralization of heterologous 169Q viruses. (A) The frequency of the 169Q (green) and 169K (orange) Env immunotypes across 28 time points from six to 206 weeks post infection. ^#^ The viruses tested in Fig 3A were isolated at the indicated time points. (B) CAP256.20 neutralization curves of wild-type (orange, 169K/M as indicated,) and 169Q mutant (green) heterologous viruses. (C) An extension of Fig 6B, the neutralization titers of the CAP256.27/20 wild-type and chimeric antibodies (from Fig 4B) were tested against the CAP256.34-wk 80 virus.
(TIF)
