# Supplementary figures and images for: Somatic hypermutation to counter a globally rare viral immunotype drove off-track antibodies in the CAP256-VRC26 HIV-1 V2-directed bNAb lineage
Source: PLoS Pathog. 2019 Sep 3;15(9):e1008005. doi: 10.1371/journal.ppat.1008005 (PMC6743783; doi:10.1371/journal.ppat.1008005)

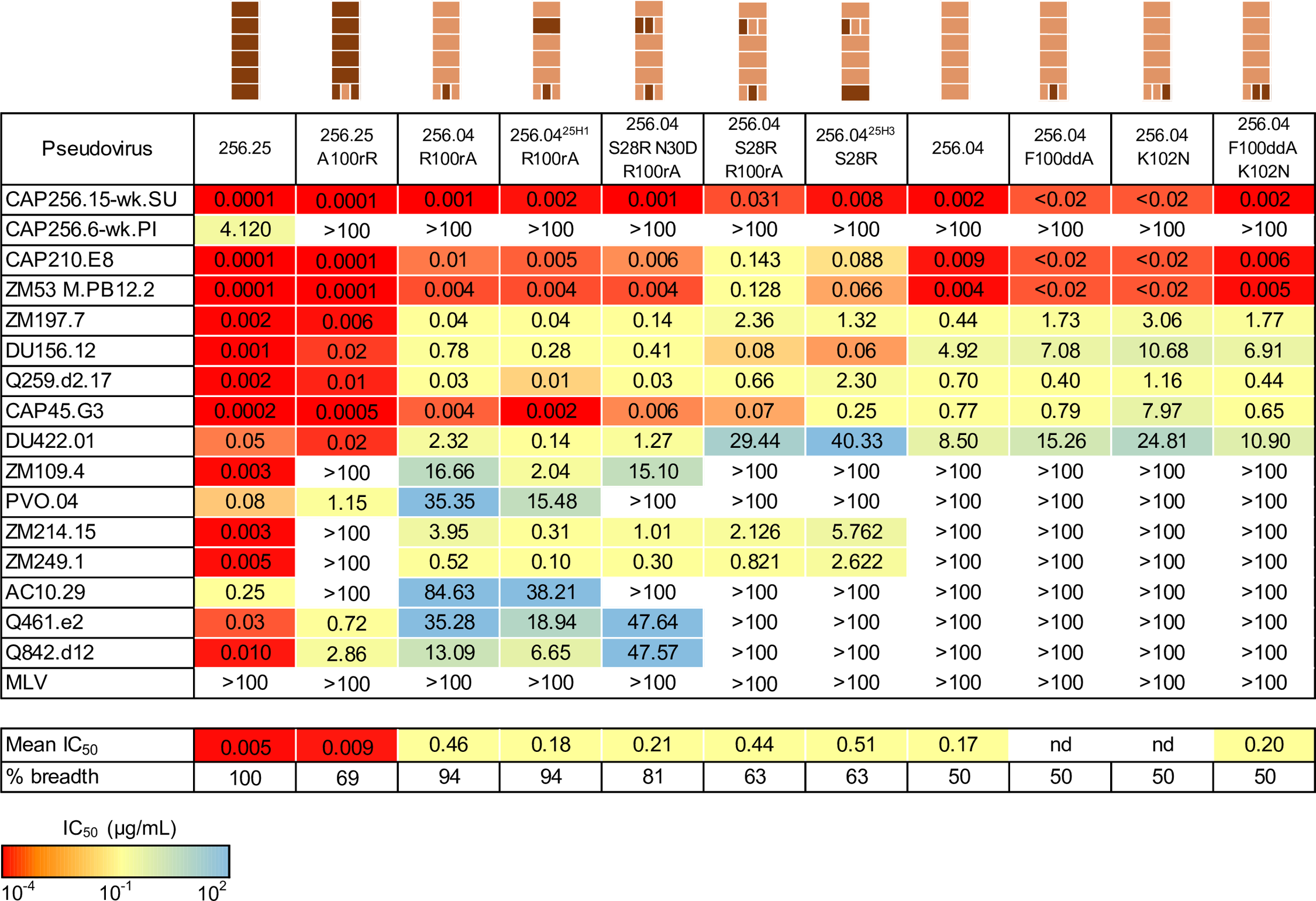

Supplement: S1 Fig — Neutralization data using mutants (indicated schematically above the table) between CAP256.25 and CAP256.04, potency indicated as per the key. Arithmetic mean titers (IC50, μg/mL) from at least two experiments are reported. (TIF) [file ppat.1008005.s001.tif]

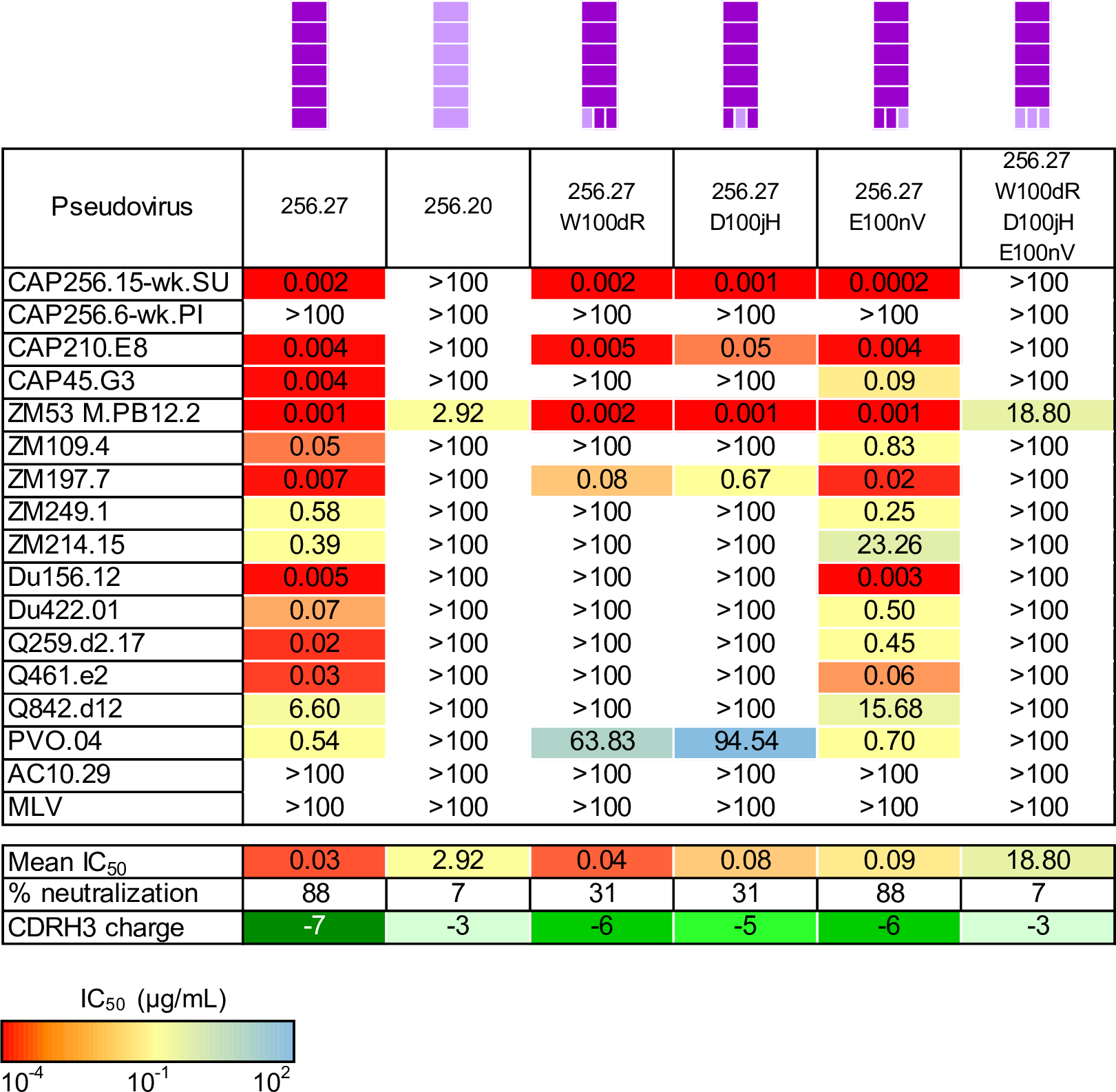

Supplement: S2 Fig — Neutralization data using mutants (indicated schematically above the table) between CAP256.27 and CAP256.20, potency indicated as per the key. Arithmetic mean titers (IC50, μg/mL) from at least two experiments are reported. (TIF) [file ppat.1008005.s002.tif]

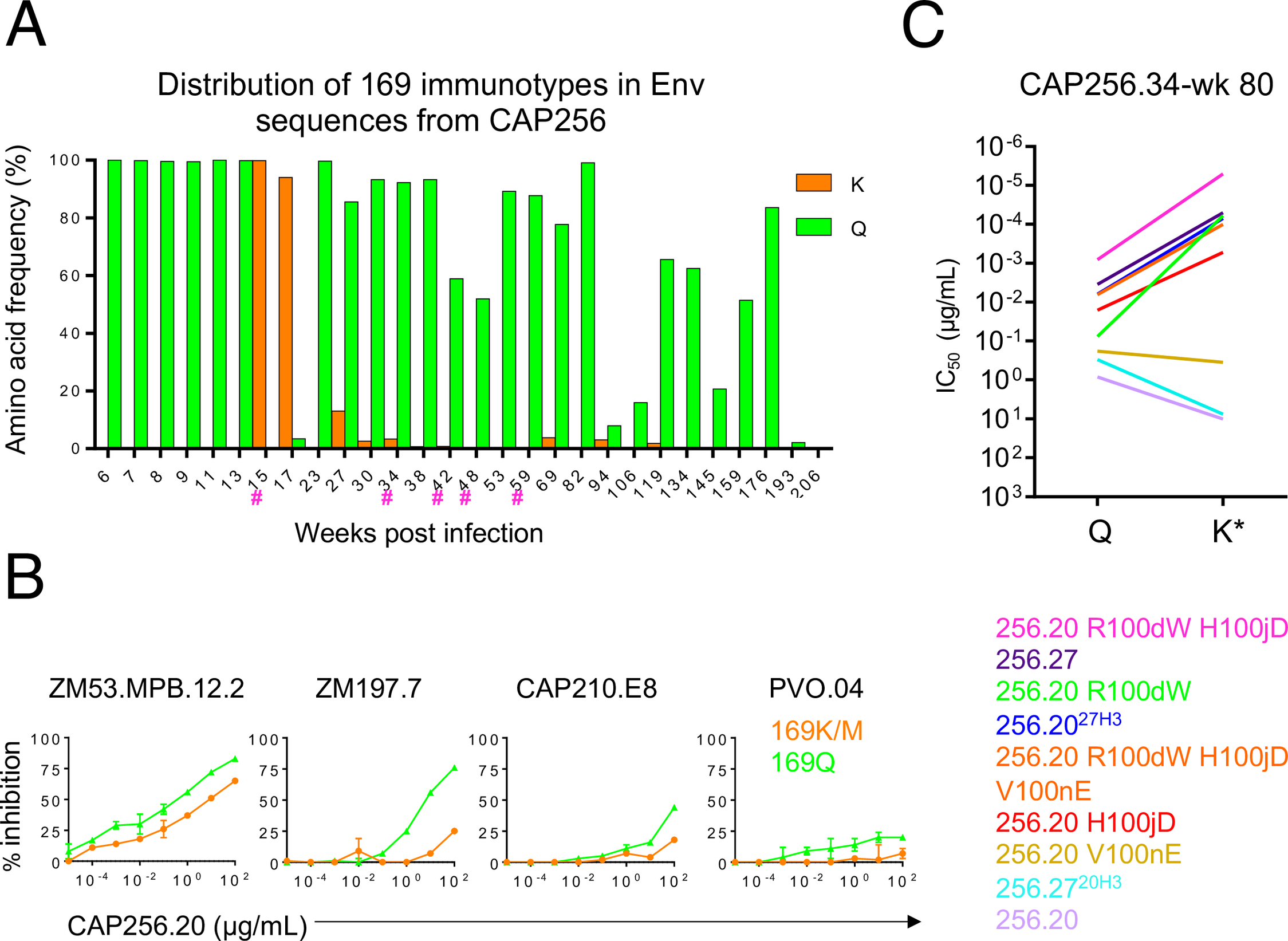

Supplement: S3 Fig — (A) The frequency of the 169Q (green) and 169K (orange) Env immunotypes across 28 time points from six to 206 weeks post infection. # The viruses tested in Fig 3A were isolated at the indicated time points. (B) CAP256.20 neutralization curves of wild-type (orange, 169K/M as indicated,) and 169Q mutant (green) heterologous viruses. (C) An extension of Fig 6B, the neutralization titers of the CAP256.27/20 wild-type and chimeric antibodies (from Fig 4B) were tested against the CAP256.34-wk 80 virus. (TIF) [file ppat.1008005.s003.tif]
